# Prevalence and Associated Risk Factors of Intestinal Parasites among Schoolchildren from Two Primary Schools in Rama Town, Northern Ethiopia

**DOI:** 10.1155/2020/5750891

**Published:** 2020-08-25

**Authors:** Yordanos Gizachew Yeshitila, Hagos Zewde, Tesfahun Mekene, Aseer Manilal, Serawit Lakew, Abinet Teshome

**Affiliations:** ^1^School of Nursing, College of Medicine and Health Sciences, Arba Minch University, Arba Minch, Ethiopia; ^2^Department of Public Health, College of Health Sciences, Aksum University, Aksum, Ethiopia; ^3^Department of Medical Laboratory Science, College of Medicine and Health Sciences, Arba Minch University, Arba Minch, Ethiopia; ^4^Department of Biomedical Science, College of Medicine and Health Sciences, Arba Minch University, Arba Minch, Ethiopia

## Abstract

**Introduction:**

Worldwide, about 3.5 billion people are affected by intestinal parasitic infections, and the majority of them are children. A perusal of the literature indicates that in Ethiopia, nearly one-third of schoolchildren are found to be infected by some sort of intestinal parasites. This study aimed to determine the prevalence of intestinal parasites among schoolchildren in Rama town in Northern Ethiopia.

**Methods:**

A school-based cross-sectional study was conducted among primary school children from two schools in Rama town during June 2017. A structured questionnaire was used to identify environmental, sociodemographic, and behavioral factors while stool specimens were collected and examined for parasites using direct wet smear with saline preparation. Data analysis was completed using the Statistical Program for Social Sciences version 24 statistical software.

**Results:**

A total of 312 school children with a mean age of 11.3 years were included. Among them, 24.4% (76) were found to be positive for at least one of the parasites. The overall infection rate was the highest among the 10–14 age groups (26.7%). Females were predominantly infected (26.7%). Altogether, eight species of intestinal parasites were identified. The most predominant protozoan and helminths were *E. histolytica*/*dispar* (10.9%) and *Schistosoma mansoni* (7.4%), respectively, and infections were mostly mono-parasitic. Coinfections with two and three intestinal parasites were identified among 13 (4.2%, [13/302]) and 2 (0.6%, [2/302]) cases, respectively. Prevalence of intestinal parasites was higher among children who did not wash their hands regularly before meals (AOR: 2.30, CI: 1.32, 4.0, *p* < 0.001) and those who frequently swam in streams (AOR: 3.12, CI: 1.07, 9.08, *p* < 0.021).

**Conclusions:**

The study revealed a high prevalence of parasitic infection and inadequate personal hygiene practices like poor handwashing and also the habit of swimming by schoolchildren in contaminated water bodies, especially the study area. To minimize the burden caused by parasitic infection, periodic deworming programs and health education should be provided to enhance the awareness of concerned participants are also warranted.

## 1. Introduction

Intestinal parasites are groups of protozoa and helminths that reside inside the human gastrointestinal tract and cause a wide range of morbidity and mortality [1]. The impact on public health by parasitic infections has been consistently underestimated in the past. However, there is now a consensus that diseases caused by intestinal parasites represent an important public health problem, especially in children [2]. Next to morbidity, intestinal parasites are associated with malnutrition, growth retardation, and physical weakness, which ultimately results in poor performance of school children [2–5]. Most instances of morbidity and mortality are caused by amoebiasis, commonly referred to as amoebic dysentery that occurs in developing and underdeveloped countries [6]. Among the protozoans, *Entamoeba histolytica* infects 500 million individuals per year, causing disease in 50 million and ultimately resulting in 100,000 deaths [7]. Likewise, *Giardia intestinalis*, a frequent causative agent of diarrhea, can also result in malabsorption in children and even retarded growth. This approximately affects a population of 200 million worldwide [5].

Helminthic infestations lead to nutritional deficiency and impaired physical development, which lead to negative consequences on cognitive function and learning ability [8, 9]. In addition to these, maternal and child health and worker productivity can also be affected by soil-transmitted helminths (STH) infections. Recent studies indicate that even moderate cases of infection may have adverse effects on growth, iron deficiency, anemia, and cognitive functions of school-age children [10].

The epidemiology of intestinal parasitic infections shows that these parasites are found in every age group, irrespective of the sex. However, incidences are higher in a certain specific area while some age groups are affected to a greater extent [9]. Studies carried out in different locales worldwide have shown that the circumstances of each individual influence the overall prevalence of intestinal parasitic infections, and children are the victims often [11–13]. Of the three STHs, hookworms account for one-third of the disease burden from all neglected tropical diseases in Sub-Saharan Africa. Out of the estimated 207 million cases of schistosomiasis, over 90% occur in Sub-Saharan Africa, with the highest prevalence among children, adolescents, and subadults [2].

Gastrointestinal diseases including those caused by intestinal parasites rank the first among communicable diseases in Ethiopia as well as in other developing countries. According to studies conducted in Ethiopia, nearly one-third of schoolchildren are found to be infected by intestinal parasites [14–16]. Studies done in different regions of the country have shown a high prevalence rate of intestinal parasites: 83.8% in a rural area close to the southeast of Lake Langano [17] and 82.4% in Zarima town located in northwest Ethiopia [18]. A similar study conducted among schoolchildren in southern Ethiopia showed an overall prevalence of 83%. The most prevalent intestinal parasite identified was the hookworm (60.2%), followed by *Schistosoma mansoni* (21.2%), *Trichuris trichiura* (14.7%), *Taenia* spp. (13.9%), *Entamoeba histolytica/dispar* (12.7%), *Ascaris lumbricoides* (6.2%), and *Giardia duodenalis* (6.2%) [19].

Intestinal parasites are endemic in several parts of Ethiopia and are an important public health problem. At the national level, intestinal parasite infections are often associated with low socioeconomic status, poor personal and environmental sanitation, overcrowding, lack of supply of safe and clean water, tropical climate, and low altitude [14–16, 20]. The prevalence and distribution of various species of intestinal parasites differ from region to region because of several environmental, social, geographical, and behavioral factors.

High prevalence of intestinal parasites is found in those with low socioeconomic statuses such as overcrowded living areas, poor environmental sanitation, improper waste disposal, unsafe water sources, and unhygienic habits. All these factors cause the severe burden of diseases and death in developing countries. As the prevalence and intensity of intestinal parasitic infections are in the peak stage among school-age children and thanks to the coordinated programs that aim this population in schools, this age group is often the first to be targeted with deworming activities. Studies on the prevalence of various intestinal parasitic infections are a prerequisite not only for the formulation of appropriate control strategies but also for predicting the levels of associated risk factors. Insofar, no similar study has been conducted in the study area, Rama town, regarding the prevalence of intestinal parasites and associated risk factors. In this background, the present study is intended to delineate the prevalence and associated risk factors of intestinal parasites among school children from two primary schools in Rama town, Northern Ethiopia.

## 2. Materials and Methods

### 2.1. Study Area, Design, Period, and Study Population

The study was carried out at two elementary schools in Rama town and the Department of Public Health, College of Health Sciences, Aksum University, which is situated in the Central Zone of the Tigray Region, Northern Ethiopia. A quantitative cross-sectional study was conducted to elucidate the prevalence and associated risk factors of intestinal parasites among school children in Rama town, Northern Ethiopia, during June 2017. Two thousand two hundred and thirty-seven students attending two primary schools during the study period were included. Students who had stayed in the *kebeles* (administrative unit) for less than a year or had taken antihelminthic drugs within a month before the time of data collection were excluded.

### 2.2. Sample Size and Sampling Procedure

The sample size was calculated using a sample size determination formula for the estimation of a single population proportion. *p* value of 0.61 was chosen from the previous study [4]. After considering 95% of confidence interval (*z* = 1.96) and 5% of marginal error (*d* = 0.05), the initial sample size was 366. Since the source population was <10,000, the sample size was amended using a correction formula and converted to 314, and finally, by computing a 10% nonresponse rate, the final sample size was consolidated as 344. To select the study subjects, they were first stratified according to their educational level (Grade 1 to Grade 8) for each school, and allocation of students was done in proportion as per the number of students in each school and their grade (level). The final sample size was fixed using the proportional allocation corresponding to each class. Finally, the study subjects were selected by a simple random sampling method using class rosters as the sampling frame.

## 3. Data and Specimen Collection

### 3.1. Specimen Collection and Laboratory Procedures

Data and specimen collections were performed with the aid of two well-trained data collectors. Prior to the data collection, the purpose of the study was lucidly explained to all participants. For the detection of parasites, ten grams of fresh stool samples was collected into screw-capped plastic containers of 100 mg capacity equipped with an applicator stick. The participants were instructed to collect sufficient samples aseptically. Collected samples were labeled and immediately transported at ambient temperature to the laboratory following appropriate safety precautions and standard operating procedures. Each stool sample was processed and examined by a direct wet mount preparation technique with saline to check the presence of cysts, trophozoites, eggs, and larvae of intestinal parasites under a light microscope at 100x and 400x magnifications [21].

### 3.2. Socioenvironmental Questionnaire

Data pertaining to the sociodemographic (age, sex, religion, level of education, family size, monthly family income, and level of education of both parents (father and mother) and environmental and behavioral factors (source of drinking water, availability of latrine in the house, handwashing habits before meals, handwashing habits after defecation, presence of dirty materials under their fingernails, the practice of nail trimming, the practice of teeth brushing, wearing shoes regularly, and habit of swimming in a stream) were solicited by face to face interview using a structured questionnaire.

### 3.3. Data Processing and Analysis

The data were analyzed using Statistical Package for Social Services (version 24). The odds ratio, 95% confidence intervals, and *p* value were calculated accordingly. Descriptive statistics such as frequencies, mean, and range were carried out to observe the distribution of the study subjects in connection with variables under study. The binary logistic regression analysis model was fitted after the cross-tabulation of each explanatory variable with the outcome variable and thereby checking the fulfillment of chi-square assumptions. Initially, bivariate analysis was carried out to select variables for multivariate analysis. Variables with *p* value <0.25 in the bivariate analysis were selected as candidates for the multivariable analysis and fitted into a logistic regression model. The *p* value of ≤0.05 was considered statistically significant.

### 3.4. Ethical Approval

The study protocol was approved by the ethical review board of Aksum University (Ref No 2204/02/17). Permission was obtained from the Mereb Leke Woreda Health Office and the Education Office and authority of the schools to conduct the study. Moreover, informed written consent was obtained from parents (guardians) of the children. Students' privacy during the interview and stool collection was maintained and the data obtained from them was kept confidential.

## 4. Results

### 4.1. Sociodemographic Characteristics

A total of 312 students from two primary schools in Rama town participated in the study, of which 146 (46.8%) were females. The mean age of the participants was 11.13 years, with an age ranging between 6 and 19. A majority of the study participants, 298 (95.5%), resided in the town while 14 (4.5%) students were residents of nearby rural villages. By religious affiliation, 278 (89.1%) were orthodox Christians and the remaining were Muslims. More than half of the study participants (53.8%) were living in a household consisting of four or five members with a mean family size of 4.87. The detailed sociodemographic characteristics of the schoolchildren are shown in Table 1.

Among the 312 samples collected, 76 (24.4%) were found to be positive for at least one of the intestinal parasites. Eight species of intestinal parasites were identified from the stool samples. The predominant protozoan parasite was *E. histolytica/dispar* which was observed in 34 (10.9%) samples followed by *G. lamblia,* found in 14 (4.5%). *Schistosoma mansoni* was the most common helminths identified from 22 (7.4%) samples followed by *Ascaris lumbricoides* 8 (2.6%) and hookworm species 7 (2.2%). One sample was positive for the ova of *Trichuris trichiura*. The overall prevalence of STH was 16% (Table 2).

The majority of the positive cases, 61 (19.6%), were infected with a mono-intestinal parasite. Bi-infections were encountered in 13 (4.2%) specimens whereas 2 (2.6%) specimens only were found to harbor a tri-parasitic infection. Only one case was found to be infected with *E. histolytica/dispar, G. lamblia*, and *A. lumbricoides* and the second one with *H. nana, E. histolytica/dispar*, and *A. lumbricoides.*

Female students showed a higher overall infection rate (26.7%) than males (22.3%). However, the difference was not statistically significant (*p* value >0.364) (Table 3). As shown in Table 2, *E. histolytica/dispar* and *G. lamblia* were relatively more prevalent among females whereas *S. mansoni* infection showed preponderance to male participants. Prevalence of individual parasites exhibited no significant differences between the two sexes except *H. nana,* which was three times more common in females than in males (Table 2). The prevalence of multiple infections among males (4.2%) and females (5.5%) was comparable.

To inspect variations among age groups, the study population was divided into 3 classes: less than or equal to 9 years, 10 to 14 years, and 15 years and above. The overall infection rate was the highest among the 10–14 years age group (26.7%), and two-thirds of children with multiple infections were found to fall in this age group. *S. mansoni* infection increased as the age increased but was not statistically significant (*p* value = 0.240). Hookworm was exclusively observed in the stool of children aged eleven years or more. The prevalence of intestinal parasites among the study participants decreased with increasing levels of education of parents while a higher proportion of rural residents (42.9%) were found to be infected compared to urban residents (23.5%). No statistically significant correlations exist among the prevalence of intestinal parasites and various sociodemographic characteristics. The distribution of intestinal parasitic infections among study subjects concerning different sociodemographic characteristics is presented in Table 3. It was found that the presence of *S. mansoni* was significantly higher among rural residents (28.6%) as compared to children who had been living in the town (7.4%) (AOR = 5.87; 95% CI (1.68, 20.48), *p* value <0.004).

### 4.2. Environmental and Behavioral Determinant Factors Associated with Intestinal Parasites

Most of the children, 300 (96.2%), used public tap water for drinking. Only 3.8% of the participants obtained water for domestic purposes from wells or streams and all of them were rural residents. Two hundred and ninety-seven students (95.2%) had latrines in their houses. Less than two-thirds, 181 (58%), of the children practiced handwashing before meals regularly while the remaining did so less frequently. Only 93 (29.8%) participants reported that they wash their hands always after defecation, while the majority of the remaining (70.2%) wash hands less frequently after defecation (54.8%) and the third group (15.4%) never remember to wash their hands after defecation. Ninety-one (29.2%) participants had the habit of nail-biting. One in every three children (33.3%) had dirt in the fingernails of their right hand. About 257 (82.4%) of the children wear shoes regularly. Nineteen students (6.1%) had the habit of swimming in streams (Table 4).

The prevalence of intestinal parasites could not be related to the source of water supply or the availability of latrines. Handwashing regularly done before meals was significantly associated with the extent of intestinal parasitic infection (AOR = 2.3, 95% CI (1.32, 4.0), *p* < 0.001). The likelihood of getting *S. mansoni* infection among students who had the habit of swimming in streams was almost three times higher compared to the cases of nonswimmers and the overall infection rate was significantly higher among frequent swimmers (AOR = 3.12; 95% CI (1.07, 9.08)). Associations of intestinal parasites with important environmental and behavioral determinant factors are presented in Table 4.

## 5. Discussion

Parasitic infection caused by protozoa and helminths is a major global health problem, especially in low-income and resource-constrained regions. Intestinal parasites are endemic in several parts of Ethiopia, and the prevalence and distribution of various species of these parasites differ from region to region and from time to time. Several studies among the school children in the country had reported a wide range of prevalence of intestinal parasites ranging from 22.7% to 83.8% among schoolchildren [17, 22]. The present study was aimed to assess the prevalence of intestinal parasites among schoolchildren in Rama town, which is a typical small town in Northern Ethiopia.

The overall prevalence of intestinal parasites in the present study is 24.4%, and it is similar to the results of studies performed in northwest Ethiopia (22.7%) [22] and from rural areas of the Kaski District of western Nepal (24.1%) [23]. Slightly higher values were reported from studies conducted among schoolchildren in Yadot (26.2%), Babile (27.2%), and Arba Minch towns (27.7%), Ethiopia, which revealed that nearly one-third of schoolchildren were infected by intestinal parasites [15, 16, 24]. However, this rate of infection was much lower than those reported from different regions of Ethiopia: Wukro town, Eastern Tigray (60.7%) (7), Dagi primary school (77.9%) [25], Azezo (72.9%) [26], and southeast of Lake Langano (83.8%) [17]. A study conducted in India had shown an increased prevalence rate of 63.9% [27] whereas even higher rates were reported from Nigeria (67.4%) [28].

Among the eight species of intestinal parasites identified, *E. histolytica/dispar* was the most common (10.9%), and this observation is similar to the studies conducted in southern Ethiopia [20] but higher than that reported from another region of the country [24]. A similar trend has been observed in previous studies conducted in Aksum (17.0%) and Wukro towns (23.2%), Ethiopia. The prevalence rate of STH (5.2%) was lower than the corresponding values reported from Zarima (23%) and Azezo towns (20%), Ethiopia [26]. Further studies done in different parts of the country showed lower prevalence rates for *A. lumbricoides* and other STHs [6, 13, 14].

In this study, *S. mansoni* infection also showed a higher prevalence rate (7.05%) as compared to the values reported in earlier studies done in different parts of Ethiopia: Babile (4.3%) [16], Aksum (1%) [14], and Wukro (3.1%) [4]. In contrast, several studies from endemic areas of Ethiopia reported higher prevalence rates ranging between 43.5% and 85% [18, 26, 29–31]. It was reported that the prevalence and intensity of *S. mansoni* infection showed the highest rates in the area well known for the dam, followed by the area around recently constructed irrigation sites and newly formed dams [32].

Among the 76 positive cases, the majority (81.2%) had only a single infection and this is in agreement with findings reported in other studies [15, 16]. The coinfection rate was 4.2% which is comparable to that observed in several studies done in Babile [16] and Arba Minch [15] but quite lower than that found in another study conducted in Dagi, Amhara National Regional State, Ethiopia [27].

Regarding the various associated factors analyzed, children who did not wash their hands before their meals were more likely to acquire an intestinal parasitic infection than their counterparts who maintained good handwashing habits (AOR 2.30; 95% CI (1.32, 4.0)). Participants who wash their hands less frequently were twice as more likely to be infected by soil-transmitted parasites (7.6% versus 3.3%). This study and its findings are similar to the work conducted in southern and northwest parts of Ethiopia [16, 26]. Some other studies reported that poor handwashing practices and fingernail hygiene provide a conducive environment for the fecal-oral transmission of intestinal parasites [24].

Overall infection rates were significantly higher among frequent swimmers (AOR: 3.12; 95% CI (1.07, 9.08)). The likelihood of getting *S. mansoni* infection among students who had the habit of swimming in streams was almost three times higher than that among nonswimmers. World Health Organization reported that lack of hygiene and frequent recreational activities in contaminated stagnant water bodies make humans susceptible to infections [33].

### 5.1. Limitations of the Study

Limitations of this study include the fact that the microscopy technique used may result in an underestimation of the prevalence of intestinal parasites in the study population. Only wet mount preparation employing normal saline was used because of the lack of reagents and financial constraints. Concentration techniques like formol-ether preparations could increase detection rates of *S. mansoni* and other helminths thereby resulting in a better estimation of the prevalence of intestinal parasites. Secondly, due to the lack of antigen tests, *Entamoeba histolytica* and *Entamoeba dispar* were not identified separately. Lastly, as the stool collection period was short, no attempt was made to investigate the parameter, seasonality, as potential seasonal fluctuations might have affected the actual prevalence.

## 6. Conclusion

The findings of the study revealed a high prevalence of intestinal parasitic infections among school children of Rama town, Ethiopia. Intriguingly, *Entamoeba histolytica*/*dispar* and *S. mansoni* were the predominant isolates in the school children. Results of risk factor analysis revealed that handwashing practices before meals and also the habit of swimming in contaminated water bodies were closely associated with the infections. Overall results imply that intestinal parasitic infections are creating a growing health problem in the study area. To minimize the burden caused by parasitic infection, periodic deworming programs and health education providing more awareness and improvement of hygiene conditions are warranted.

## Figures and Tables

**Table 1:** Sociodemographic characteristics among schoolchildren in the two involved schools in Rama town, Northern Ethiopia.

Sociodemographic characteristics		Number (*n*)	Percent
Age group (years)			
≤9		89	28.5
10–14		195	62.5
15+		28	9.0

Sex			
Male		166	53.2
Female		146	46.8

Religion		
Christian		278	89.1
Muslim		34	10.9

Class level		
1–3		116	37.2
4–6		96	30.8
7-8		100	32.0

Family size		
≤5		213	68.3
6+		99	31.7

Residence		
Urban		298	95.5
Rural		14	4.5

Mother's educational status		
Unable to read and write		89	28.5
Primary school		162	51.9
Secondary school		50	16.1
College and above		11	3.5

Father's educational status		
Unable to read and write		52	16.6
Primary school		145	46.5
Secondary school		92	29.5
College and above		23	7.4

Total		312	100

This study was carried out in June 2017.

**Table 2:** Distribution of intestinal parasites according to the sex in the two primary schools in Rama town, Northern Ethiopia.

Parasite species	Male (*n* = 167)	Female (*n* = 146)	Total (*n* = 312)^*∗*^
	Positive (%)	Positive (%)	Positive (%)
*E. histolytica/dispar*	13 (7.8)	21 (14.4)	34 (10.9)
*G. lamblia*	6 (3.6)	8 (5.5)	14 (4.5)
*S. mansoni*	14 (8.4)	8 (5.5)	22 (7.4)
*A. lumbricoides*	5 (3.0)	3 (2.1)	8 (2.6)
Hookworm species	4 (2.4)	3 (2.1)	7 (2.2)
*H. nana*	1 (0.6)	3 (2.1)	4 (1.3)
*E. vermicularis*	1 (0.6)	2 (1.4)	3 (1)
*T. trichiura*	1 (0.6)	0 (0)	1 (0.3)

*Note.* ^*∗*^The sum of the columns is greater than the total number of infected children because of the coinfections. This study was carried out in June 2017.

**Table 3:** Prevalence of intestinal parasitic infection stratified by sociodemographic characteristics among schoolchildren in two primary school children in Rama town, Northern Ethiopia, June 2017.

Sociodemographic characteristics	Intestinal parasites		*p* value	COR (95% CI)	AOR (95% CI)
	Negative (%)	Positive (%)			
Age group (years)					
≤9	70 (78.7)	19 (21.3)	0.690	1.250 (0.42, 3.72)	
10–14	143 (73.3)	52 (26.7)	0.322	1.670 (0.60, 4.63)	
15+	23 (82.1)	5 (17.9)		1	

Sex					
Male	129 (74.8)	37 (22.3)		1	
Female	107 (73.3)	39 (26.7)	0.364	0.79 (0.47, 1.32)	

Religion					
Christian	208 (74.8)	70 (25.2)	0.471	1.57 (0.62, 3.95)	
Muslim	28 (82.4)	6 (17.6)		1	

Grade					
1–3	83 (71.6)	33 (28.4)	0.363	1.33 (0.72, 2.47)	
4–6	76 (79.2)	20 (20.8)	0.714	0.88 (0.45, 1.74)	
7–8	77 (77.0)	23 (23.0)		1	

Family size					
≤5	167 (78.4)	46 (21.6)		1	1
6+	69 (69.2)	30 (30.8)	0.097	1.57 (0.92, 2.70)	1.37 (0.75, 2.50)

Residence					
Urban	228 (76.2)	70 (23.5)		1	1
Rural	8 (57.1)	6 (42.9)	0.109	2.44 (0.82, 7.28)	1.16 (0.22, 5.97)

Mother's educational status					
Unable to read and write	62 (69.7)	27 (30.3)	0.409	1.96 (0.39, 9.68)	
Primary school	123 (76.9)	39 (24.1)	0.658	1.43 (0.29, 6.88)	
Secondary school	42 (84)	8 (16.0)	0.860	0.86 (0.15, 4.73)	
College ad above	9 (81.8)	2 (18.2)		1	

Father's educational status					
Unable to read and write	33 (63.5)	19 (36.5)	0.105	2.73 (0.81, 9.24)	2.66 (0.73.84)
Primary school	11 (217.2)	33 (22.8)	0.565	1.40 (0.44, 4.40)	1.75 (0.51, 6.02)
Secondary school	72 (78.3)	20 (21.7)	0.647	1.32 (0.40, 4.32)	1.85 (0.51, 6.75)
College and above	19 (82.6)	4 (17.4)		1	1

**Table 4:** Association of environmental and behavioral determinant factors with the prevalence of intestinal parasites among schoolchildren in Rama town, Northern Ethiopia, June 2017.

Determinant factor	Intestinal parasitic infection *N* = 76 (%)	*p* value	COR (95% CI)	AOR (95% CI)
Source of water				
Pipe water	73 (24.3)		1	
Well/streams	3 (25.0)	0.958	1.04 (0.27, 2.93)	

Latrine availability				
Yes	70 (23.6)		1	1
No	6 (40.0)	0.157	2.16 (0.64, 7.44)	0.93 (0.18, 4.88)

Handwashing before feeding				
Always	38 (20.3)		1	
Sometimes	38 (30.4)	0.001	2.35 (1.39, 3.98)	2.30 (1.32, 4.0)

Handwashing after defecation				
Always	19 (20.4)		1	1
Sometimes	45 (26.3)	0.288	1.39 (0.75, 2.55)	
Not at all	12 (25.0)	0.535	1.30 (0.57, 2.96)	

Habit of nail-biting				
No	48 (21.7)		1	
Yes	28 (30.8)	0.092	1.61 (0.93, 2.77)	1.38 (0.76, 2.50)

Dirty materials in fingernails				
Present	30 (28.8)	0.193	1.43 (0.84, 2.44)	0.89 (0.49, 1.63)
Absent	46 (22.1)		1	

Wearing shoes				
Sometimes	19 (34.5)	0.055	1.85 (0.99, 3.47)	1.32 (0.65, 2.64)
Always	57 (22.2)		1	1

Habit of swimming in streams				
No	68 (23.1)		1	1
Yes	8 (42.1)	0.021	3.04 (1.18, 7.78)	3.12 (1.07, 9.08)

COR: crude odd ratio; AOR: adjusted odd ratio.

## Data Availability

The datasets supporting the conclusions of this article are included within the article and the supplementary file. For further queries, the datasets used and/or analyzed during the current study are also available from the corresponding author upon reasonable request.

## Abbreviations

IPI: Intestinal parasitic infection
STHs: Soil-transmitted helminths
SOPs: Standard operating procedures.

## Supplementary Materials

Supplementary Materials The supplementary file contains a questionnaire for the assessment prevalence and associated factors of intestinal parasites among schoolchildren in Rama town, Northern Ethiopia, June 2017, in English and Tigrigna version and standard form for recording the results of fecal examinations.
