# Supplementary material for: Prevalence and Associated Risk Factors of Intestinal Parasites among Schoolchildren from Two Primary Schools in Rama Town, Northern Ethiopia
Source: Can J Infect Dis Med Microbiol. 2020 Aug 25;2020:5750891. doi: 10.1155/2020/5750891 (PMC7468605; doi:10.1155/2020/5750891)
Supplement: Supplementary Materials — The supplementary file contains a questionnaire for the assessment prevalence and associated factors of intestinal parasites among schoolchildren in Rama town, Northern Ethiopia, June 2017, in English and Tigrigna version and standard form for recording the results of fecal examinations. [file 5750891.f1.doc]

## Annex A: Questionnaire (English version)

**Questionnaire for the assessment Prevalence and Associated Factors of Intestinal** **Parasites among Schoolchildren in Rama Town, Northern Ethiopia; June 2017**

You are kindly requested to respond each question with an answer that best describes you. Information you provide will be used only for the study and will never be transferred to third part directly or indirectly. For the sake of confidentiality, your name will not be mentioned in this questionnaire. You are free to consider your participation and to withdraw from the study at any point in the course if you wish to do so.

Thank you in advance

**Identification Number**____________ **School**______________

1. **Socio-demographic data**
   1. Age (in years) ____________
   2. Sex
2. Male
3. Female
   1. Grade _______
   2. Religion
4. Christian
5. Muslim
6. Other (specify)_________
   1. Number of family members___________
   2. Monthly family income (ETB)__________
   3. Residence
7. Urban
8. Rural
   1. Mother’s educational status
9. Unable to read and write
10. Able to read and write
11. Attended school (Mention highest level)
    1. Father’s educational status
12. Unable to read and write
13. Able to read and write
14. Attended school (Mention highest level)
15. **Environmental conditions**
    1. Do you have a toilet in your house
16. Yes
17. No
    1. Where do you get water for drinking?
18. Tap water
19. Well
20. Stream/river
21. Other (specify)____________
22. **Behavioral factors**
    1. Do you wash your hands before eating?
23. No
24. Yes
    1. If yes, how often
25. Sometimes
26. Always
    1. Do you wash your hands after defication?
27. No
28. Yes
    1. If yes, how often
29. Sometimes
30. Always
    1. Do you trim your finger nails using your teeth?
31. No
32. Yes
    1. Do you wear shoes?
33. No
34. Yes
    1. If yes, how often?
35. Sometimes
36. Always
    1. Is there dirty material in finger nails of the right hand (by observation)
37. No
38. Yes

## Annex B: Questionnaire (Tigrigna version)

**ኣብ ራማ ቀዳማይ ብርኪ ኣብያተ ትምህርቲ ብዛዕባ ፅግዕተኛ ሓሳ¢: ንዝግበር መፅናዕቲ ዝተዳለወ ቃለ መሕትት**

ንሕድሕድ ሕቶ ናዓኻ/ኺ ብትትክክል ዝገልፅ መልሲ ንክትህበለይ/ብለይ ብትሕትና ይሓትት:: ንሕድሕድ ሕቶ እትህቦ/ብዮ መልሲ ነዚ መፅናዕቲ ጥራሕ ዘገለግል እንትኾን ንማንም ሳልሳይ ኣካል ብቀጥታ ይኹን ብተዘዋዋሪ ዘይወሃብ ም£<ኑ ኣቀዲመ ከፍልጥ ይፎቱ:: ምሽጥራዊነቱ ንምሕላው ሽምካ/ኪ ኣብዚ ቃለመሕትት ዘይግለፅ እንትኾን መንነትካ/ኪ ካብ ናይ ሰገራ ናሙና ምስ ዝግበር ምርመራ ውፅኢት ንምትሕሓዝ ብግላዊ መለለዪ ቁፅሪ ክግለፅ እዩ:: ኣብዚ መፅናዕቲ ዘለካ/ኪ ተሳትፎ ኣብ ደስ ዝበለካ/ኪ ግዜ ናይ ምቁራፅ መሰልካ/ኪ ዝተሓለወ እንትኾን ንእትገብሮ/ሪዮ ተሳትፎ የመስግን::

**መለለዪ ቑፅሪ** ------------------ **ሽም ቤት ትምህርቲ**_____________________________________ **ዕለት**________________

1. ግላዊ መረዳእታ
   1. ዕድመ ----------
   2. ፆታ

1. ተባዕታይ

2. ኣንስታይ

- 1. ደረጃ ትምህርቲ-----------
  2. ሃይማኖት

1. ክርስትያን

2. ሙስሊም

3. ካሊእ (ይገለፅ)______________

1. ናይ ቤተሰብ መረዳእታ
   1. በዝሒ ኣባላት ስድራ-------------
   2. ወርሓዊ እቶት ስድራ (ብብር)_______________
   3. ናይ ኣዶ ደረጃ ትምህርቲ

1. ኣይተምሃረትን

2. ምፅሓፍን ምንባብን ትኸእል

2. ስሩዕ ትምህርቲ ተማሂራ (ዝለዓለ ደረጃ ይገለፅ)--------------

- 1. ናይ ኣቦ ደረጃ ትምህርቲ

1. ኣይተምሃረን

2. ምፅሓፍን ምንባብን ይክእል

3. ስሩዕ ትምህርቲ ተማሂሩ (ዝለዓለ ደረጃ ይገለፅ)--------------

- 1. አብ ገዛኩም ሽቓቅ ኣለኩምዶ?

1. ኣለና

2. የብልናን

- 1. ዝስተ ማይ ካበይ ትጥቀም/ሚ?

1. ካብ ቡምባ

2. ካብ ዕላ

2. ካብ ገረብ

4. ካሊእ(ይገለፅ)----------------

1. ናይ ግሊ ንፅህና
   1. ቅድሚ ምግቢ ምምጋብካ/ኪ እድካ/ኪ ትሕፀበ/ቢ ዶ?

1. ኣይሕፀብን

2. ሓሓሊፉ

3. ኩሉ ግዜ

- 1. ድሕሪ ሽቃቅ ምጥቃምካ/ኪ እድካ/ኪ ትሕፀብ/ቢ ዶ?

1. ኣይሕፀብን

2. ሓሓሊፉ ይሕፀብ

3. ኩሉ ግዜ ይሕፀብ

- 1. ፅፍሪ ኣፃብዕትካ/ኪ ብስንካ/ኪ ትቆርፅ/ፂ ዶ?

1. ኣይቆርፅን

2. ሓሓሊፉ

3. ኩሉ ግዜ

- 1. ሳእኒ ትጥቀም/ሚ ዶ?

1. ኣጥቀምን

2. ሓሓሊፉ ይጥቀም

3. ኩሉ ግዜ ይጥቀም

3.5. ኣብ ፅፍሪ የማናይ ኢድ ርስሓት ኣሎ ዶ? (ብምርኣይ ዝምለስ)’

1. እወ

2. የለን

**ሽም ኣካቢ መረዳእታ_______________________**

**ፊርማ____________** **ዕለት_____________________**

## Annex C. Standard form for recording the results of faecal examinations

|  |  | Date ___/___/______ |
| --- | --- | --- |
| **Personal data** |  |  |
| ID number_________ | School______________ |  |
| Age_________ (years) | Sex M___F ____ |  |
| **Stool examination** | **Result** | |
| **Helminthes** | Positive | Negative |
| *Ascaris lumbricoides* |  |  |
| *Trichuris trichiura* |  |  |
| *Hymenolepis nana* |  |  |
| Hook worm species |  |  |
| *Enterobius vermicularis* |  |  |
| *Schistosoma mansoni* |  |  |
| *Strongloides stercoralis* |  |  |
| Taenia species |  |  |
| Other/s (specify) |  |  |
| **Protozoa** |  |  |
| *Entamoeba histolytica/dispar* |  |  |
| *Giardia lamblia* |  |  |
| Other/s (specify) |  |  |
